# Vulnerabilidad, cronicidad y envejecimiento: la importancia de integrar la experiencia con la medicación de las personas mayores en la Atención Farmacéutica

**DOI:** 10.23938/ASSN.1092

**Published:** 2024-12-20

**Authors:** Martha Milena Silva-Castro

**Affiliations:** Olivet Farmacia Granollers Barcelona España; Sociedad Española de Optimización de la Farmacoterapia (SEDOF) Granollers Barcelona España; Medicines Optimisation Systems Granollers Barcelona España

Sra. Editora:

Existen múltiples formas de medir el impacto de las diversas intervenciones farmacéuticas que pueden, y deben, llevarse a cabo para disminuir la morbimortalidad que ocasionan los numerosos fármacos utilizados por las personas ingresadas en servicios de Geriatría. Al margen de la falta de unificación de los procedimientos que suelen denominarse *atención farmacéutica*, lo realmente relevante es advertir sobre los riesgos ocasionados a las personas mayores por la polimedicación sin seguimiento ni acompañamiento especializado, y que agravan su fragilidad^1^.

En el número 45 de la revista Anales del Sistema Sanitario de Navarra, publicado en 2022, Raquel Marín-Gorricho y col^2^ presentaron su investigación titulada *Impacto de la atención farmacéutica en pacientes polimedicados ingresados en un servicio de agudos de Geriatría*, donde mostraron una alta prevalencia de tratamientos inadecuados en personas ancianas ingresadas. Durante los 16 meses del periodo de estudio, incluyeron 218 pacientes con una duración media de ingreso hospitalario de 10,9 días (DE: 8,3), el 26,6% de los cuales tenían prescritos más de diez medicamentos. La intervención farmacéutica realizada por estos investigadores consistió, primeramente, en una revisión de la historia clínica y de las prescripciones de fármacos utilizando la segunda versión en castellano de los criterios STOPP/START^3^ para detectar y estimar la prevalencia de prescripciones potencialmente inadecuadas (PPI) y problemas relacionados con la medicación (PRM). Con esta información elaboraron un informe que también incluía acciones para solucionarlos, acciones que fueron propuestas por la farmacéutica hospitalaria al geriatra responsable de la persona durante su ingreso. Posteriormente cuantificaron la diferencia entre el número de PPI y PRM al ingreso y al alta hospitalaria. Si la modificación de los tratamientos supuso un cambio ≥75% en la prevalencia de cada PPI y PRM, se consideró aceptada la propuesta farmacoterapéutica.

Como señalaron los propios autores del estudio^2^, la aplicación de los criterios STOPP/START está en expansión, debido principalmente a que ofrecen formas concretas, sistemáticas y viables de mejorar la prescripción en estos grupos complejos de pacientes. Esta afirmación ha sido corroborada por la publicación de una tercera versión en español^4^ que amplía la difusión de estos criterios en colectivos hispanoparlantes. Esta versión es especialmente útil en polimedicación porque recoge una lista actualizada de medicamentos potencialmente inapropiados y, además, menciona posibles omisiones durante la prescripción, particularmente en situaciones de polifarmacia y multimorbilidad. Añade 76 criterios a los 114 criterios de la segunda versión, alcanzando los 190 criterios (57 START y 133 STOPP), que se organizan por sistemas y están alineados con la revisión de la medicación. Aunque Marín-Gorricho y col no pudieron utilizar estos criterios actualizados por la fecha de realización de su estudio^2^, todas las versiones han recopilado recomendaciones consensuadas por geriatras y otros especialistas que atienden a personas mayores, haciéndolos aplicables en clínica. No obstante, estos criterios son tan extensos y exhaustivos que su aplicación práctica puede ser complicada. Para facilitar este proceso, Marín-Gorricho y col^2^ emplearon la herramienta de apoyo *CheckTheMeds*®, ya que ha demostrado gran utilidad para apoyar la toma de decisiones clínicas por parte de equipos de profesionales de la salud^5^. Sin embargo, la complejidad de la medicación de pacientes mayores hace necesarias herramientas más adaptadas a las circunstancias particulares de cada persona, preferiblemente centradas en su experiencia con la medicación^6^, y en un abordaje integral que considere un cuidado biopsicosocial en geriatría^7^.

Los resultados de Marín-Gorricho y col^2^, con rangos de frecuencias de PPI generalmente semejantes a los de otras investigaciones que aplican los criterios STOPP/START en su primera y segunda versión, siguen demostrando que estos criterios metodológicos son limitados para abordar la complejidad del paciente geriátrico polimedicado. Estos criterios omiten aspectos relevantes como la expectativa de vida, la plurimorbilidad, la fragilidad y, sobre todo, la toma de decisiones por parte del paciente y -por ende- de sus cuidadores^8^. Las personas con limitaciones crónicas tienen peores experiencias al recibir atención hospitalaria, y los cuestionarios que simplifican estos factores múltiples pueden no resultar lo suficientemente sensibles para reflejar aspectos importantes de la experiencia de los pacientes^9^. Por ello, en cualquier medición del impacto de una intervención sanitaria sería imprescindible considerar los modelos de decisiones compartidas con los pacientes que, con el paso de los años, se han estado ejecutando con un enfoque integral y donde la coordinación se está revelando como la clave del éxito^10^. En el caso de personas mayores tratadas por cuadros agudos, debería considerarse la coordinación no solo con el personal de Farmacia y Geriatría que los atiende, sino con sus cuidadores directos, aunque la persona se encuentre hospitalizada para tratar una condición aguda. Las personas cuidadoras juegan un rol fundamental^11^ y, en términos prácticos, coordinarse con ellas desde el ingreso facilitaría la integración con los grupos de apoyo a los que pueden recurrir en atención primaria al alta hospitalaria. Las personas allegadas al adulto mayor se convierten en sus cuidadores durante la transición entre niveles asistenciales^12^, desarrollando funciones de conciliación para coordinar las adaptaciones en sus tratamientos hechas desde el hospital y trasladarlas a la vida domiciliaria. Por tanto, estas cuidadoras requieren comprender el porqué de los tratamientos, qué medicamentos deberían ser continuados o descontinuados al salir del ingreso, y cómo utilizarlos en el domicilio del paciente. Esta coordinación puede contribuir a la gestión de la atención personalizada, a apoyar la autogestión y a promover la prescripción social^10^.

La intervención farmacéutica de Marín-Gorricho y col^2^ implicó comunicación entre personal de Farmacia y Geriatría, pero no mencionaron ningún tipo de entrevista o comunicación directa con pacientes o sus cuidadores. Este hecho revela que pueden presentarse omisiones en las intervenciones de esta investigación al no considerar el punto de vista del paciente ni sus experiencias, ni las particularidades de su contexto sociosanitario al no consultar con cuidadores o familiares sobre la viabilidad de las intervenciones farmacéuticas. Es necesario reconocer que hay contextos sanitarios en los que persisten lagunas de conocimiento y que requieren mejorar la calidad de las intervenciones, evaluaciones y presentación de informes^13^. Estandarizar la medición y presentación de informes, de indicadores y de resultados de prescripción podría facilitar la extracción de datos útiles comparativos de poblaciones y entornos heterogéneos. También es importante seguir investigando formas de reducir el impacto negativo de las barreras interprofesionales y de la falta de comunicación con cuidadores en los resultados de cualquier intervención en geriatría^9^^,^^14^. Tanto profesionales como tomadores de decisiones deben comprender mejor los contextos en los que se ha realizado cada intervención y los mecanismos que conducen a su éxito^9^. Por este motivo, en contextos de polimedicación, cronicidad y envejecimiento, comprender la experiencia con la medicación de los pacientes es uno de sus principales principios de una verdadera optimización de la medicación^15^^,^^16^ que ayude efectivamente a los pacientes a obtener los mejores resultados de sus tratamientos.

Para comprender la experiencia con la medicación (aproximación personal del paciente cuando toma medicamentos, conformada por la suma de todos los eventos e implicaciones que tiene a lo largo de su vida^17^) se deben analizar las actitudes, expectativas, preocupaciones, comprensión, motivaciones, creencias y comportamientos de las personas sobre sus tratamientos^17^. Representa el eje de la Gestión Integral de la Medicación porque conocer esta experiencia implica relacionarse con las personas mediante una escucha activa, respeto y calidez, a través de una relación continua de confianza entre el paciente y su farmacéutico^18^. Cuando se efectúa este cuidado y acompañamiento de persona a persona, se confirma que esta experiencia con la medicación está contextualizada^19^ y obedece a una fuerza cultural^20^, debido a que no solamente se trata de una experiencia corporal, simbólica e intencional, sino que es relacional e intersubjetiva^21^. En función del aprendizaje y de las vivencias referidas a los otros, esta experiencia situada se construye socialmente porque lleva implícitas las creencias individuales y colectivas, la cultura, la realidad sociosanitaria, económica y política de cada persona en su contexto.

Teniendo en cuenta todas estas consideraciones, la aplicación de los criterios STOPP/START como intervención farmacéutica debería complementarse con la Gestión Integral de la Medicación^22^, preferiblemente mediante Unidades de Optimización de la Farmacoterapia, como realizaron Palchik y col^23^ en un estudio en el contexto argentino semejante al de Marin-Gorricho y col^2^. Estos autores confirmaron que seguir los principios de la optimización de la medicación^15^^,^^16^ y considerar la experiencia de pacientes adultos mayores con su medicación es esencial, no solo por su fragilidad^1^^,^^22^ y la complejidad de sus tratamientos, sino por las consecuencias clínicas, económicas y humanísticas de los problemas de salud producidos por los medicamentos, aún más graves en colectivos vulnerables como este.
